# Protective function of albiflorin against ferroptosis in exhaustive exerciseinduced myocardial injury via the AKT/Nrf2/HO-1 signaling

**DOI:** 10.1590/acb393524

**Published:** 2024-08-12

**Authors:** Zhuang Tian, Zhenyu Li

**Affiliations:** 1 Zhengzhou University – College of Physical Education – Zhengzhou, Henan Province, China.; 2 Jeonbuk National University – College of Natural Science – Department of Sport Science – Jeonju, South Korea.; 3 Sias University – Xinzheng, Henan Province, China.

**Keywords:** Biological Products, Exercise, Ferroptosis, Heart

## Abstract

**Purpose:**

It has been reported that exhaustive exercise (EE) causes myocyte injury, and eventually damages the function of the myocardia. Albiflorin (AF) has anti-inflammatory, antioxidant, and anti-apoptosis effects. In this study, we determined whether AF could mitigate the EE-induced myocardial injury and research the potential mechanisms.

**Methods:**

The rat model of EE was built by forced treadmill running method. Rats were intraperitoneally injected with AF before EE once daily for one week. The relative factors levels were examined by commercial kits. The apoptosis was appraised using a TdT-mediated dUTP nick end labeling assay kit. The ACSL4, GPX4, Nrf2, pAKT/AKT, and HO-1 contents were assessed by western blot.

**Results:**

AF lessened EE-induced cardiac myocytes ischemic/hypoxic injury and reduced the contents of myocardial injury biomarkers in the serum. AF lessened EE-induced cardiac myocyte apoptosis, inflammatory response, oxidative stress, and ferroptosis in myocardial tissues. However, the influences of AF were overturned by the co-treatment of AF and LY294002. AF activated the AKT/Nrf2/HO-1 signaling pathway in myocardial tissues in vivo.

**Conclusions:**

AF could curb cardiac myocytes ferroptosis, thus diminishing the EE-induced myocardial injury through activating the AKT/Nrf2/HO-1 cascade.

## Introduction

As we all know, physical exercise is very important to human health^1^. Proper physical activity can diminish the incidence of cardiovascular and cerebrovascular illnesses and reduce the risk of death in patients with respiratory diseases, hypertension, and diabetes^2^^–^ 4. However, studies have shown that exhaustive exercise (EE) is not necessarily good for health and can even have adverse effects on the body, like heart^5^. As a result, many scholars have recently focused their attention on the damage caused by EE. Studies have shown that EE can lead to myocardial damage and myocardia dysfunction, which increases the risk of connected cardiac disease^6^^,^ 7. In addition, there is increasing evidence that EE causes myocardial injury, which is related to myocardial oxidative stress, apoptosis, inflammatory reaction, and ferroptosis^8^^–^ 10. Nevertheless, the precise mechanism of EE and myocardial injury remains indistinct.

Albiflorin (AF) is the main glycoside in the root of Peony, Ranunculaceae. Studies have found that AF has anti-inflammatory, antioxidant and neuroprotective effects^11^. Liu et al.^12^ exposed that AF could relieve neuropathic pain and comorbid anxiety-like behavior caused by chronic systolic injury of the sciatic nerve by inhibiting NLRP3 inflammasome activity. Besides, Zhang et al.^11^ revealed that AF lessened inflammatory reaction and oxidative stress through modulating the NF-κB/NLRP3 signaling pathway in methotrexate-tempted enteritis. Moreover, Li et al.^13^ confirmed that AF had neuroprotective effects and was involved in regulating metabolic disorders and inhibiting neuroinflammation. However, there are limited data on the influence of AF preconditioning on myocardial injury induced by EE, and the relevant mechanisms are indeterminate.

Herein, we conjectured that AF might be operative to ameliorate myocardial injury after EE. In this research, the rat model of EE was built by forced treadmill running method. The aims of this study were to obtain a thorough understanding of the influences of AF on EE-induced myocardial injury and explore the potential mechanisms.

## Methods

### Animals

This research was approved by the Ethical Committee of College of Physical Education, Zhengzhou University (Ethical NO. 2022003). The adult male Sprague Dawley (SD) rats (10 weeks old) were obtained from the Beijing Vital River Laboratory Animal Technology Co., Ltd. (Beijing, China). These rats were fed at 22 ± 2°C with 45–55% relative humidity and 12-h light/dark cycle, and freely access to food and water.

### Experimental design and animal modeling

The SD rats were randomly assigned to six groups (n = 6):

- Control (Ctrl) group;
- EE group;
- EE + low dose of AF (L-AF) group;
- EE + middle dose of AF (M-AF) group;
- EE + high dose of AF (H-AF) group;
- EE + AF + LY294002 (AF + LY) group.

Albiflorin (Sigma-Aldrich, St. Louis, MO, United States of America) was dissolved in normal saline. The rats in L-AF, M-AF, and H-AF groups were intraperitoneally injected with 5, 10, and 20 mg/kg AF at 1 h before EE every day for one week, respectively. AF + LY group was intraperitoneally injected with 20 mg/kg AF and 10 mg/kg Akt pathway inhibitor LY294002 (GenePharma, Shanghai, China) at 1 h before EE per day for one week. At the same time, the control and EE groups were injected with equal volume of normal saline once a day for one week.

The rat model of EE-induced myocardial injury was constructed in accordance with a previous paper^14^. Before modeling, a motorized treadmill (Shenzhen Bride Biotechnology Co., Ltd., Shenzhen, China) was used to train rats adaptively daily for seven consecutive days at a rate of 15 m/min. Using a treadmill with a 10° gradient, the rats were subjected to daily uphill running for a period of seven days following initial training. At first, the animals ran at 12 m/min, increasing their speed by 3 m/min every 5 min until they reached 27 m/min. When rats exhaust themselves, they become unable to run despite mild electroshock, which is defined as the sign of exhaustion. Following this, they rested for 3 min before repeating the above procedure three times. Using the objective sign of exhaustion, this exercise regimen was repeated for seven days.

In the sedentary control group, rats were confined to resting conditions on a treadmill (speed 0 m/min). At 24 h after EE modeling experiment, all rats were anesthetized by intraperitoneal injection of pentobarbital sodium (50 mg/kg). Whereafter, serum and left ventricular myocardial tissues from each group were collected. Part of the myocardial tissue was fixed in formaldehyde (10%; Sigma) for preparation of paraffin sections, and the other part was stored at -80°C for protein extraction.

### Hematoxylin-eosin staining

The myocardial tissues of rats were coped with 4% formalin solution (Sigma) for 8 h. Next, these tissues were exposed to 70% ethanol solution (Sigma) for 5 min, and treated with 80, 90, 95%, and absolute ethanol to carry out gradient dehydration. Whereafter, the myocardial tissues were exposed to xylene (Sigma) for 30 min, and then embedded in paraffin (Sigma). The section thickness was 3 μm, and 10 pieces of each specimen were taken for continuous hematoxylin-eosin (HE) detection.

### Evaluation of myocardial injury biomarkers in serum

The serum samples were gained from each group rats. The creatine kinase (CK) and cardiac troponin I (cTnI) levels in serum were assessed by exploiting enzyme-linked immunosorbent assay (ELISA) kits (Ruixin Biotech, Quanzhou, China) in line with the protocol. Additionally, the levels of creatine kinase myocardial band isoenzyme (CK-MB) and lactate dehydrogenase (LDH) were detected by utilizing an automated biochemical analyzer (Olympus, Tokyo, Japan).

### TdT-mediated dUTP nick end labeling assay

TdT-mediated dUTP nick end labeling (TUNEL) in situ cell death detection kit (Sigma) was employed to evaluate apoptosis. In brief, after deparaffinization, rehydration, permeabilization, and blocking, the myocardial tissue sections were exposed to TUNEL reaction mixture (50 μL; Sigma) at 37°C for 1 h. Then, converter-POD (50 μL; Sigma) was added on the sections and hatched for 0.5 h. Next, the sections were coped with DAB (50 μL; Sigma). Finally, the sections were stained with DAPI (Sigma) and observed through a microscopy (Olympus). Besides, the caspase-3 activity in myocardial tissue was evaluated by exploiting a caspase-3 activity assay kit (Beyotime, Shanghai, China).

### ELISA assay

The serum samples were homogenized and centrifuged to gather the supernatant. The interleukin (IL)-6 (Ruixin Biotech), tumor necrosis factor-α (TNF-α; Ruixin Biotech), IL-1β (Ruixin Biotech), and IL-18 (Ruixin Biotech) ELISA kits were exploited to assess the IL-6, TNF-α, IL-1β, and IL-18 contents according to the directions.

### Evaluation of MDA, GSH, SOD, and ROS levels

The myocardial tissues of rats were lysed, and the lysate was centrifuged for assessing the levels of malondialdehyde (MDA) (Nanjing Jiancheng Bioengineering Institute, Nanjing, China), glutathione (GSH) (Nanjing Jiancheng Bioengineering Institute), superoxide dismutase (SOD) (Nanjing Jiancheng Bioengineering Institute), and reactive oxygen species (ROS) (Nanjing Jiancheng Bioengineering Institute) by commercial kits. The kit’s instructions were used for specific experimental procedures.

### Iron assay

With the aim of assessing the iron concentration in myocardial tissues of rats, an iron assay kit (Abcam) was applied according to the instructions.

### Western blot

The myocardial tissues were lysed, and BCA protein assay kit (Solarbio, Beijing, China) was exploited for protein quantification. The nuclear proteins were extracted by employing nuclear extraction kit (NCM Biotech, Suzhou, China). Then, the proteins (50 μg per hole) were segregated by 12% SDS-PAGE gel and transferred onto PVDF film (Solarbio). The film was blocked and probed with primary antibodies at 4°C. The antibody as follow: anti-ACSL4 (ab155282; 1:2,000; Abcam, Cambridge, MA, United States of America), anti-GPX4 (ab125066; 1:1,000; Abcam), anti-Nuclear Nrf2 (#33649; 1:1,000; Cell Signaling Technology, Beverly, MA, United States of America), anti-Lamin B (#13435; 1:1,000; Cell Signaling Technology), anti-Akt (ab179463; 1:3,000; Abcam), anti-p-Akt (ab18206; 1:1,000; Abcam), anti-HO-1 (ab305290; 1:1,000; Abcam), anti-GAPDH (AF7021; 1:3,000; Affinity, Changzhou, China). Then, the films were coped with goat anti-rabbit IgG (Affinity) for 1 h. The bands were observed by exploiting a chemiluminescence kit (Sigma) and investigated via Image J software (NIH, Bethesda, MD, United States of America).

### Statistical assay

The data analysis was conducted utilizing GraphPad Prism 8.0 (La Jolla, CA, United States of America). The data were presented as means ± standard deviation. One-way analysis of variance was used to examine the significant differences between groups. Statistically significant was considered when *p* < 0.05.

## Results

### Albiflorin mitigated exhaustive exercise-induced myocardial injury

**Figure 1:**
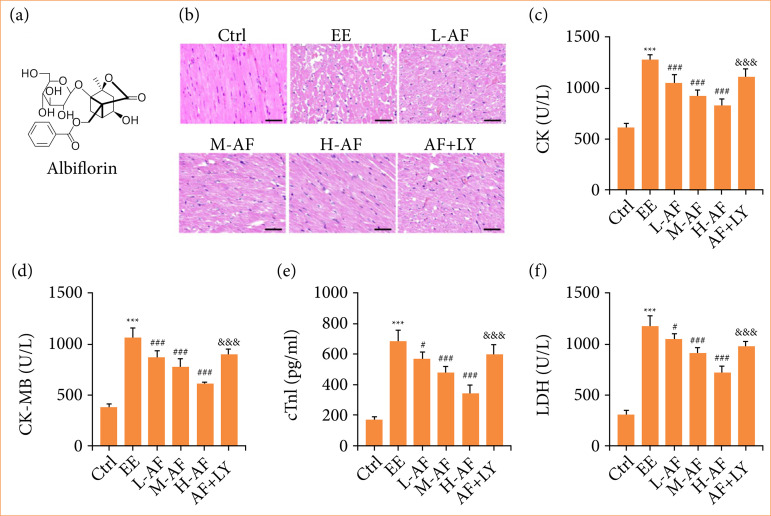
AF regulated EE-induced myocardial injury. **(a)** The chemical structure of AF. **(b)** Representative eosin hematoxylin staining of cardiac myocytes were shown. Scale bar: 50 μM. **(c–f)** The contents of CK, CK-MB, cTnI, and LDH were examined by commercial kits.

Source: Elaborated by the authors. AF: albiflorin; Ctrl: control; EE: exhaustive exercise; CK: creatine kinase; CK-MB: creatine kinase myocardial band isoenzyme; cTnI: cardiac troponin I; LDH: lactate dehydrogenase; ***p < 0.001, versus the Ctrl group; ^#^ *p* < 0.05, ^##^ *p* < 0.01, ^###^ *p* < 0.001, versus the EE group; ^&&&^ *p* < 0.001 versus the H-AF group.

Firstly, we appraised the effects of AF in myocardial tissues with AF treatment. The chemical structure of AF was exposed in Fig. 1a. For HE staining, normal cardiac myocytes were stained pink, and ischemic/hypoxic cardiac myocytes were stained red because of their higher acidophilia. The staining of cardiac myocytes in Ctrl group displayed clear boundaries, uniform pink cytoplasm, and blue-violet nuclei. However, we exposed that EE group showed obvious myocardial ischemic/hypoxic alterations, with lots of ischemic/hypoxic cardiac myocytes that were stained red, indistinct cardiac myocyte boundaries. There was a marked diminution in ischemic/hypoxic alterations in L-AF, M-AF, and H-AF groups, and the effect of reduction was more obvious with high dose of AF treatment.

The interesting thing is that the effect of AF on the cardiac myocytes was abolished when an amalgamation of AF and LY294002 was applied (Fig. 1b). Besides, the contents of CK, CK-MB, cTnI, and LDH in serum were utilized to assess myocardial injury following EE treatment. As displayed in Figs. 1c–1f, the EE group rats had apparently raised contents of CK, CK-MB, cTnI, and LDH versus the Ctrl group. Compared to group EE, the CK, CK-MB, cTnI, and LDH levels were abridged after AF treatment in a dose-dependent mode, while this influence was lessened by combined therapy of AF and LY294002. These results suggested that AF lessened EE-induced cardiac myocytes ischemic/hypoxic injury and reduced the contents of myocardial injury biomarkers in the serum.

### Albiflorin mitigated cardiac myocytes apoptosis induced by exhaustive exercise

**Figure 2:**
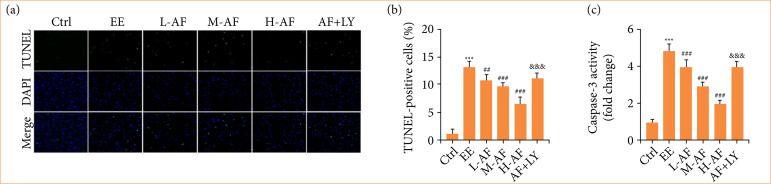
AF mitigated EE-induced cardiac myocytes apoptosis. **(a** and **b)** The apoptosis was appraised by TUNEL assay. **(c)** The caspase-3 activity was examined by commercial kits.

Source: Elaborated by the authors. AF: albiflorin; Ctrl: control; EE: exhaustive exercise; ***p < 0.001, versus the Ctrl group; ^##^ *p* < 0.01, ^###^ *p* < 0.001, versus the EE group; ^&&&^ *p* < 0.001 versus the H-AF group.

Next, we examined the effect of AF on cardiac myocytes apoptosis. We found that the cardiac myocytes apoptosis (Figs. 2a and 2b) and caspase-3 activity (Fig. 2c) of rats were heightened by EE induction versus the group Ctrl, while these effects were diminished after AF treatment in a dose-dependent mode. However, the anti-apoptotic influences of AF were overturned by the co-treatment of AF and LY294002. These consequences exposed that AF receded the apoptosis of cardiac myocytes post EE.

### Albiflorin lessened exhaustive exercise-induced inflammatory response

**Figure 3:**
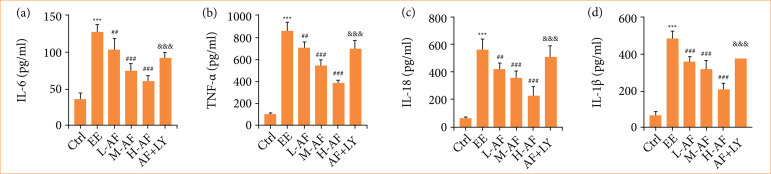
AF lessened EE-induced inflammatory response. The contents of **(a)** IL-6, **(b)** TNF-α, **(c)** IL-18, and **(d)** IL-1β were examined by ELISA kits.

Source: Elaborated by the authors. AF: albiflorin; Ctrl: control; EE: exhaustive exercise; IL: interleukin; TNF-α: tumor necrosis factor α; ELISA: enzyme-linked immunorbent assay; ***p < 0.001, versus the Ctrl group; ^##^ *p* < 0.01, ^###^ *p* < 0.001, versus the EE group; ^&&&^ *p* < 0.001, versus the H-AF group.

With the purpose of discovering the influence of AF on EE-induced inflammatory response in rats, we conducted ELISA assay to measure the contents of IL-6, TNF-α, IL-1β, and IL-18. We observed that these pro-inflammatory factors levels in serum (Figs. 3a–3d) of rats were remarkably amplified by EE induction with respect to group Ctrl. In comparison with group EE, the IL-6, TNF-α, IL-1β, and IL-18 levels were reduced after AF treatment in a dose-dependent mode, whereas this result was reduced by LY294002 co-treatment. These consequences revealed that AF apparently diminished EE-induced inflammatory response in serum of rats.

### Albiflorin curbed exhaustive exercise-induced oxidative stress in myocardial tissues

**Figure 4:**
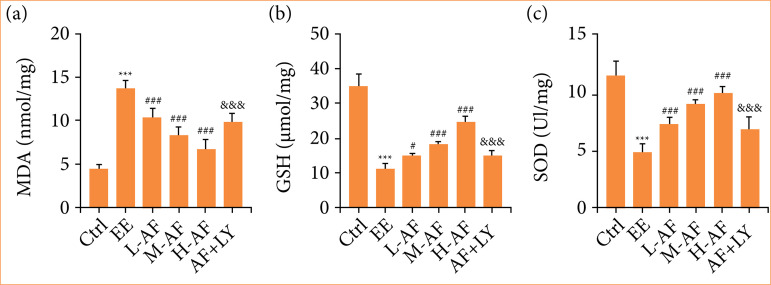
AF restrained EE-induced oxidative stress in myocardial tissues. The contents of **(a)** MDA, **(b)** GSH, and **(c)** SOD were examined by commercial kits.

Source: Elaborated by the authors. AF: albiflorin; Ctrl: control; EE: exhaustive exercise; MDA: malondialdehyde; GSH: glutathione; SOD: superoxide dismutase; ***p < 0.001, versus the Ctrl group; ^#^ *p* < 0.05, ^###^ *p* < 0.001, versus the EE group; ^&&&^ *p* < 0.001, versus the H-AF group.

We inspected the effects of AF on oxidative stress of myocardial tissues. We exposed that the abundance of MDA (Fig. 4a) in myocardial tissues were boosted by EE induction, while this consequence was impaired after AF treatment in a dose-dependent mode. With respect to group Ctrl, the GSH (Fig. 4b) and SOD (Fig. 4c) contents were impaired after EE treatment. In comparison with group EE, the GSH and SOD levels were enhanced after AF treatment in a dose-dependent mode. The interesting thing was that the anti-inflammatory influences of AF were neutralized by the co-treatment of AF and LY294002. Therefore, we demonstrated that AF abridged oxidative stress in myocardial tissues with AF treatment.

### Albiflorin restrained exhaustive exercise-induced ferroptosis in myocardial tissues

**Figure 5:**
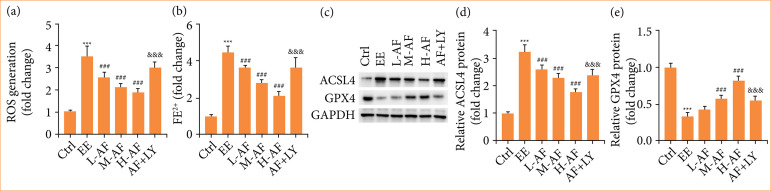
AF restrained EE-induced ferroptosis in myocardial tissues. **(a)** The abundance of ROS was evaluated by commercial kit. **(b)** The iron content was scrutinized by commercial kit. **(c–e)** The ACSL4 and GPX4 levels were assessed by western blot.

Source: Elaborated by the authors. AF: albiflorin; Ctrl: control; EE: exhaustive exercise; ROS: reactive oxygen species; ***p < 0.001, versus the Ctrl group; ^##^ *p* < 0.01, ^###^ *p* < 0.001, versus the EE group; ^&&^ *p* < 0.01, ^&&&^ *p* < 0.001, versus the H-AF group.

We examined the effects of AF on ferroptosis of myocardial tissues. The contents of ROS (Fig. 5a) and Fe^2+^ (Fig. 5b) in myocardial tissues were heightened by EE induction, however this result was weakened after AF co-treatment in a dose-dependent mode. Successively, western blot was carried out to evaluate the abundance of ferroptosis-related proteins. The ACSL4 abundance (Figs. 5c and 5d) was boosted, but GPX4 level (Figs. 5c and 5e) was reduced in myocardial tissues after EE induction, while these effects were diminished after AF co-treatment in a dose-dependent mode. Nevertheless, the anti-ferroptosis influences of AF were neutralized by joint therapy of AF and LY294002. In this part, these results uncovered that AF repressed ferroptosis in myocardial tissues with EE induction.

### Albiflorin activated the AKT/Nrf2/HO-1 signaling pathway

**Figure 6:**
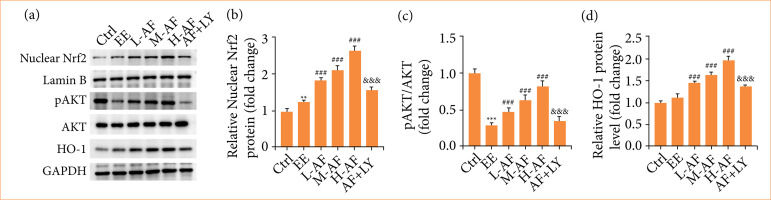
AF activated the AKT/Nrf2/HO-1 signaling pathway. **(a–d)** The contents of Nrf2, pAKT/AKT, HO-1 were appraised by Western blot.

Source: Elaborated by the authors. AF: albiflorin; Ctrl: control; EE: exhaustive exercise; **p < 0.01, ***p < 0.001, versus the Ctrl group; ^##^ *p* < 0.01, ^###^ *p* < 0.001, versus the EE group; ^&&&^ *p* < 0.001, versus the H-AF group.

We confirmed whether AF modulated EE-induced myocardial injury by AKT/Nrf2/HO-1 signaling pathway. We revealed that the contents of nuclear Nrf2 protein (Figs. 6a and 6b) and HO-1 (Figs. 6a and 6d) in myocardial tissues were enhanced after EE induction versus group Ctrl. With respect to group EE, the nuclear Nrf2 and HO-1 levels were amplified by AF treatment in a dose-dependent mode, while this effect was apparently overturned by combined therapy of AF and LY294002. On the contrary, the pAKT/AKT ratio (Fig. 6a and 6c) in myocardial tissues were abridged by EE induction relative to group Ctrl. However, the pAKT/AKT ratio in myocardial tissues were enhanced by AF treatment in a dose-dependent mode, but it declined after AF and LY294002 co-treatment. Hence, these conclusions confirmed that AF activated the AKT/Nrf2/HO-1 signaling pathway in myocardial tissues in vivo.

## Discussion

Studies have shown that EE-induced myocardial injury is closely related to a variety of biological processes^15^. Loboda et al.^16^ have shown that oxidative stress triggered by the overproduction of ROS could promote the activation of apoptosis signaling pathways and play a key role in EE-induced myocardial injury. In addition, Deng et al.^17^ observed significantly boosted contents of apoptosis-linked regulatory factors in impaired myocardial tissues.

Apoptosis was shown to be the main mechanism to decipher the connection between EE and subsequent pathological changes. Moreover, Suzuki et al.^18^ exposed that EE-induced inflammatory responses have been displayed to be extremely enhanced along with boosted inflammatory cytokines after cardiac dysfunction, suggesting a potential mechanism for EE-tempted myocardial injury. Additionally, Xiao et al.^10^ revealed that ferroptosis occurred in cardiac myocytes of mice after EE induction. Furthermore, EE can cause permanent damage to human health and body^19^^,^ 20. The above mechanisms provide another direction for the therapy of myocardial damage after EE.

Ke et al.^21^ confirmed that AF could relieve myocardial ischemia caused by isoproterenol treatment in rats. In this paper, we exposed that AF lessened EE-induced cardiac myocytes ischemic/hypoxic injury and reduced the contents of myocardial injury biomarkers (CK, CK-MB, cTnI, and LDH) in the serum, which was alike with the studies of Ke et al.^21^. We also found that the influence of AF was lessened by combined therapy of AF and LY294002. Besides, Zhu et al.^22^ found that, in ischemia-reperfusion rat model, AF abridged apoptosis of brain tissues, declined the contents of IL-1β, IL-6, and TNF-α, and mitigated oxidative stress. Herein, we revealed that AF receded the apoptosis and caspase-3 activity of cardiac myocytes post-EE treatment. AF apparently diminished the pro-inflammatory factors and oxidative stress levels in serum of rats post-EE treatment. However, the anti-apoptotic, anti-inflammatory, and antioxidative influences of AF were overturned by the co-treatment of AF and LY294002. These conclusions were in agreement with Zhu et al.’s study^22^.

Ferroptosis is a particular form of cell death, which was linked with MDA accretion and iron-overloading. Ferroptosis is positively regulated by ACSL4 and negatively regulated by GPX4^23^^,^ 24. Dong et al.^25^ found that Nrf2 could negatively modulate ferroptosis by regulating GPX4 levels. In this study, we firstly uncovered that AF repressed ferroptosis in myocardial tissues with EE induction, while the anti-ferroptosis influences of AF were curbed by combined therapy of AF and LY294002. Nrf2 is a key transcription factor tacks part in modulating oxidative damage^26^. Additionally, He et al.^27^ unfolded that the activation of Nrf2 signaling curbed the oxidative stress. Moreover, recent studies have found that the activation of Nrf2/HO-1 pathway could reduce ferroptosis^28^^,^ 29. Zhu et al.^22^ also found that AF could activate Nrf2/HO-1 pathway. Furthermore, Jeong et al.^30^ exposed that AF could modulate thermogenic genes through PI3K/AKT pathway. Herein, we confirmed that AF activated the AKT/Nrf2/HO-1 signaling pathway in myocardial tissues *in vivo*.

In this paper, we confirmed for the first time that AF could alleviate EE-induced myocardial injury. However, there were still some defects in this paper. We only completed the study in animal models, which still needs to be further verified in clinical practice.

## Conclusion

AF significantly decreased EE-induced cardiac myocytes ischemic/hypoxic injury and curbed the contents of myocardial injury biomarkers in the serum, concomitantly with alleviation of myocardial apoptosis. Furthermore, AF reduced oxidative stress injury and ferroptosis in hearts of rats that were subjected to EE treatment. AF pretreatment induced the activation of the AKT/Nrf2/HO-1 pathway in vivo. In conclusion, AF protected rat heart against EE-induced myocardial injury at least partially via activating the AKT/Nrf2/HO-1 signaling pathway. Our findings confirmed that AF could potentially be developed as a therapeutic drug for myocardial injury induced by EE.

## Data Availability

The datasets used and/or analyzed during the current study are available from the corresponding author on reasonable request.
